# The Complete Mitochondrial Genomes of Six Heterodont Bivalves (Tellinoidea and Solenoidea): Variable Gene Arrangements and Phylogenetic Implications

**DOI:** 10.1371/journal.pone.0032353

**Published:** 2012-02-23

**Authors:** Yang Yuan, Qi Li, Hong Yu, Lingfeng Kong

**Affiliations:** Fisheries College, Ocean University of China, Qingdao, Shandong, China; The Centre for Research and Technology, Hellas, Greece

## Abstract

**Background:**

Taxonomy and phylogeny of subclass Heterodonta including Tellinoidea are long-debated issues and a complete agreement has not been reached yet. Mitochondrial (mt) genomes have been proved to be a powerful tool in resolving phylogenetic relationship. However, to date, only ten complete mitochondrial genomes of Heterodonta, which is by far the most diverse major group of Bivalvia, have been determined. In this paper, we newly sequenced the complete mt genomes of six species belonging to Heterodonta in order to resolve some problematical relationships among this subclass.

**Principal Findings:**

The complete mt genomes of six species vary in size from 16,352 bp to 18,182. Hairpin-like secondary structures are found in the largest non-coding regions of six freshly sequenced mt genomes, five of which contain tandem repeats. It is noteworthy that two species belonging to the same genus show different gene arrangements with three translocations. The phylogenetic analysis of Heterodonta indicates that *Sinonovacula constricta*, distant from the Solecurtidae belonging to Tellinoidea, is as a sister group with *Solen grandis* of family Solenidae. Besides, all five species of Tellinoidea cluster together, while *Sanguinolaria diphos* has closer relationship with *Solecurtus divaricatus*, *Moerella iridescens* and *Semele scaba* rather than with *Sanguinolaria olivacea*.

**Conclusions/Significance:**

By comparative study of gene order rearrangements and phylogenetic relationships of the five species belonging to Tellinoidea, our results support that comparisons of mt gene order rearrangements, to some extent, are a useful tool for phylogenetic studies. Based on phylogenetic analyses of multiple protein-coding genes, we prefer classifying the genus *Sinonovacula* within the superfamily Solenoidea and not the superfamily Tellinoidea. Besides, both gene order and sequence data agree that *Sanguinolaria* (Psammobiidae) is not monophyletic. Nevertheless, more studies based on more mt genomes via combination of gene order and phylogenetic analysis are needed to further understand the phylogenetic relationships in subclass Heterodonta.

## Introduction

Mitochondrial DNA (mtDNA) is the only extranuclear genome in animal cytoplasm [1]. Most metazoan mitochondrial genomes are covalently closed circular molecules which range from 14 to 42 kb in length [2], but see [3]. The typical mitochondrial genome contains the same 37 genes: 13 for protein subunits of oxidative phosphorylation enzymes (*atp6*, *atp8*, *cox1–3*, *cob*, *nad1–6* and *nad4l*), two for mitochondrial ribosomal RNAs [small and large subunit ribosomal RNA (*rrnS* and *rrnL*)] and 22 for the transfer RNA genes (tRNAs) genes necessary for translating these 13 proteins [4]. In general, there are few intergenic nucleotides except for a single large non-coding region generally thought to contain elements that control the initiation of replication and transcription [4]. Owing to abundance of mitochondria in cells, lack of recombination, maternal inheritance (except for [5]), absence of introns, and higher evolutionary rates, mtDNA sequences are extensively used for comparative and evolutionary genomics, molecular evolution, population genetics, species identification, and phylogenetic relationships at various taxonomic levels [6]–[9].

For some phyla of animal, mitochondrial gene arrangements seem seldom to have changed. With few notable exceptions, those vertebrates studied, for instance, have identical gene arrangements [10]. However, mollusks, especially bivalves, which display an extraordinary amount of variation in gene arrangement, challenge this rule. Gene arrangement has been shown to be very powerful characters for reconstructing evolutionary relationships, and the rapidity of rearrangement within a lineage determines the level at which rearrangements are likely to be phylogenetically informative [10], [11].

In recent studies, phylogenetic analysis based on complete mt sequence data have proved to enhance resolution and statistical confidence of inferred phylogenetic trees when compared with analyses based only on small portions of the mtDNA [12]–[15]. With technological and methodological advances, and associated decreasing costs of DNA sequencing, the amplification and sequencing of whole mt genomes has become routine [16]. Consequently, there have been significant increases in the number of complete mitochondrial sequences available during the last ten years. Nevertheless, to date, only ten complete mitochondrial genomes of Heterodonta, which is by far the most diverse major group of Bivalvia [17], have been determined.

Heterodonta, encompassing richly speciose families such as the Cardiidae, Tellinidae, Veneridae and Lucinidae, and including major economic groups such as clams, cockles, geoducks and razor shells, can be hugely abundant in both marine and freshwater systems, and of considerable ecological importance in community structure as well as a trophic resource [17]–[19]. However, taxonomy and phylogeny of Heterodonta are long-debated issues, and a complete agreement has not been reached yet, even if this subclass has a rich fossil history extending from the Lower Palaezoic, with major radiations in the Late Mesozoic [20]–[22]. In particular, the morphologically-inferred phylogenies of subclass Heterodonta were challenged by recent phylogenetic studies based on molecular data. For example, the Gastrochaenidae and Hiatellidae do not form a monophyletic group with the other families of the order Myoida [23]–[24]. Monophyly of the Lucinoidea is not supported, with the families Thyasiridae and Ungulinidae not closely related to the Lucinidae [25].

The superfamily Tellinoidea of the subclass Heterodonta consists of five families (Tellinidae, Donacidae, Psammobiidae, Semelidae, Solecurtidae) [26]. Based on the information of paleontology and morphology of Tellinoidea, large numbers of research works have been performed to study the evolutionary history and taxonomy within this superfamily over a long time [27]–[30]. Howbeit, compared with molecular analyses carried out to investigate relationships within individual families of Heterodonta (*e.g.*, Veneridae [31], [32], Sphaeriidae [33], Thyasiridae [34]), there have been few attempts to make comprehensive analysis of phylogenetic relationships of Tellinoidea on molecular level so far, not to mention analysis based on complete mitochondrial genome.

In this paper, we newly sequenced the complete mt genomes of six heterodont bivalves, including five from four families (Tellininae, Psammobiidae with two species, Semelidae, Solecurtidae) within superfamily Tellinoidea and one from superfamily Solenoidea which was ever classified in Tellinoidea, and compared their different gene arrangements. In addition, the six newly determined sequences, together with the heterodont mt genomes available in GenBank, were used to recover the phylogeny of Heterodonta in order to resolve some problematical relationships among this subclass.

## Results and Discussion

### Genome composition

The main structural features of the six newly sequenced mt genomes in this study are summarized in Table 1. The complete mitochondrial genomes of MOIR-0101 *Moerella iridescens* [JN398362], SADI-0111 *Sanguinolaria diphos* [JN398363], SAOL-0112 *Sanguinolaria olivacea* [JN398364], SESC-0121 *Semele scaba* [JN398365], SICO-0201 *Sinonovacula constricta* [JN398366] and SODI-0131 *Solecurtus divaricatus* [JN398367] vary in size from 16,352 bp (*S. diphos*) to 18,182 bp (*S. olivacea*). Length differences are mostly in virtue of the variation in tandem repeats within the non-coding region. The placement of all coding genes on the same strand and the lack of one protein coding gene *atp8* are the most distinctive features of marine bivalve mt genomes [35], [36], without exceptions for six studied species. The overall A+T content of six newly sequenced complete mt genomes ranges from 59.19% (*S. scaba*) to 67.08% (*S. constricta*). In addition, overlapping genes, which are presumably to help prevent rearrangements of gene order and loss of genes during evolution in mammalian [37], are a common phenomenon in all newly sequenced mt genomes (Table 1).

**Table 1:** Main structural features of the six newly sequenced mt genomes in this study.

	*Moerella* *iridescens*	*Sanguinolaria* *diphos*	*Sanguinolaria* *olivacea*	*Semele* *scaba*	*Sinonovacula* *constricta*	*Solecurtus* *divaricatus*
**Total size**	16799	16352	18182	17117	17224	16749
**A+T %**	65.72	63.36	65.27	59.19	67.08	60.15
***rrnS***	863	876	949	861	909	887
***rrnL***	1268	1343	1346	1330	1228	1380
**No. of tRNA**	22	21	21	20	22	22
**No. of gene** **overlapping**	3	6	9	2	2	4
**Size range of** **gene overlapping**	1	1 to 44	1 to 44	1 to 20	1 to 3	1
***cox1***	1671	1755	1758	1692	1692	1725
	(ATA/TAA)	(ATA/TAG)	(ATA/TAA)	(ATG/TAA)	(ATT/TAA)	(ATA/TAG)
***cox2***	861	873	858	1206	843	867
	(ATG/TAG)	(ATG/TAG)	(ATG/TAA)	(ATG/TAG)	(ATG/TAG)	(ATG/TAA)
***cox3***	867	936	936	894	804	894
	(ATA/TAA)	(ATG/TAG)	(ATA/TAG)	(ATG/TAG)	(ATG/TAG)	(ATG/TAG)
***nad1***	924	924	939	921	930	927
	(GTG/TAG)	(ATT/TAG)	(ATA/TAA)	(ATG/TAG)	(ATA/TAG)	(ATA/TAA)
***nad2***	1014	1071	1077	1056	1056	1062
	(ATA/TAA)	(ATG/TAG)	(ATG/TAA)	(TTG/TAA)	(ATG/TAG)	(ATG/TAG)
***nad3***	360	363	378	360	366	360
	(ATT/TAA)	(ATT/TAA)	(ATG/TAG)	(ATA/TAG)	(ATG/TAG)	(ATG/TAA)
***nad4***	1335	1303	1362	1365	1392	1347
	(ATA/TAG)	(ATA/T)	(ATA/TAA)	(GTG/TAG)	(ATG/TAA)	(TTG/TAG)
***nad4L***	291	315	312	291	288	291
	(ATG/TAG)	(ATG/TAA)	(ATG/TAA)	(GTG/TAA)	(ATG/TAA)	(GTG/TAG)
***nad5***	1746	1788	1800	1737	1761	1734
	(TTG/TAA)	(ATA/TAA)	(ATT/TAG)	(ATG/TAA)	(ATT/TAA)	(ATG/TAG)
***nad6***	537	567	555	633	531	567
	(ATT/TAA)	(ATA/TAG)	(ATA/TAG)	(ATA/TAA)	(ATG/TAG)	(ATA/TAG)
***cob***	1233	1233	1242	1176	1146	1248
	(ATA/TAA)	(ATG/TAG)	(ATG/TAG)	(ATT/TAG)	(ATG/TAA)	(GTG/TAA)
***atp6***	777	720	687	711	702	846
	(ATG/TAA)	(ATA/TAG)	(ATG/TAA)	(ATG/TAA)	(ATA/TAG)	(ATG/TAA)

For each, total size of the mt genome, the percent of overall A+T content, size of *rrnS* and *rrnL*, number of tRNA, number of gene overlapping and its size range, and size of the protein coding genes (start and stop codons in parentheses) are presented. Gene lengths are in bp.

### Protein coding genes

Table 1 shows the initiation and termination codons for the 12 protein-coding genes (PCGs) encoded by the six mt genomes. Most of PCGs (64/72) appear to start with the conventional codon ATN (ATG, *N* = 34; ATA, *N* = 22; ATT, *N* = 8), which is typical for metazoan mt genomes [2]. There are also TTG (*N* = 3) and GTG (*N* = 5) acted as start codons, which are not unusual start codons in molluscan mt genomes but in several gastropod mt genomes [38]. All 12 PCGs of six mt genomes end in full termination codon (TAG, *N* = 37; TAA, *N* = 34), except for *nad4* gene of *S. diphos* ending with the incomplete stop codon T which may be modified to a complete TAA stop codon via posttranscriptional polyadenylation [39]. In contrast to the available heterodont bivalves mt genomes from GenBank, the mt genome of *S. olivacea* has the longest *cox1* (1758 bp) and *nad2* (1077 bp) genes, *S. scaba* has the longest *cox2* (1206 bp) and *nad6* (633 bp) genes, and *S. diphos* has the shortest *nad4* (1303 bp) gene.

### Transfer and ribosomal RNA genes

In the mt genomes of metazoan, almost all amino acids but leucine and serine are decoded by only one tRNA each [40]. Without exception, there are 22 typical tRNAs interspersed throughout the mt genome of *M. iridescens*, *S. divaricatus*, and *S. constricta*. The *trnF* is missing in both *S. diphos* and *S. olivacea*, and *S. scaba* lacks *trnY* and *trnS1*. Deficiencies of tRNA genes are often observed in protozoans, fungi, algae, plants and low metazoans [41], [42]. In this study, most of tRNAs can be folded into the typical secondary structures (not shown).

BLAST searches indicated approximate locations of the two rRNA genes, but their exact boundaries can not been determined [43]. The size of *rrnL* flanked by *nad6* and *atp6* in all six mt genomes ranges from 1228 bp (*S. constricta*) to 1380 bp (*S. divaricatus*). The length of *rrnS* varies from 861 bp (*S. scaba*) to 949 bp (*S. olivacea*). The *rrnS* genes of *M. iridescens*, *S. diphos* and *S. divaricatus* position in between *trnG* and major non-coding region. However, *rrnS* genes of *S. olivacea*, *S. scaba*, and *S. constricta* lie in between *cox2* and *trnS1*, between *trnW* and *cox2*, and between *trnM* and *cox3*, respectively. Both lengths of *rrnL* and *rrnS* are within the range of genome sizes of already sequenced molluscan mtDNAs.

### Non-coding regions

There are a large number of non-coding regions (NCR) including in the six mt genomes each. The number of NCR varies from 16 (*S. diphos* and *S. olivacea*) to 25 (*S. constricta*). The total length of unassignable nucleotides ranges from 1022 bp (6.25% of the genome) in *S. diphos* to 2730 bp (15.01% of the genome) in *S. olivacea* (Table 2).

**Table 2:** A comparison of non-coding regions (NCR) within the six mt genomes.

				Largest NCR		
Species	No. of NCR	Total lenth (bp)	Proportion of themt genome (%)	Lenth (bp)	A+T %	Location
*Moerella iridescens*	23	1604	9.55	1200	70.00	*rrnS - trnM*
*Sanguinolaria diphos*	16	1022	6.25	674	70.77	*rrnS - trnM*
*Sanguinolaria olivacea*	16	2730	15.01	2272	69.67	*trnG* - *trnI*
*Semele scaba*	23	1567	9.15	1166	58.92	*cob* - *trnG*
*Sinonovacula constricta*	25	2134	12.39	1492	66.89	*nad2* - *trnK*
*Solecurtus divaricatus*	22	1160	6.93	775	65.81	*rrnS - trnM*

Due to lacking discrete conserved sequence blocks, the control regions of invertebrates' mt genomes, unlike those of vertebrates, are not well characterized [44]. In general, the mt genome contains one major non-coding region with some peculiar patterns (e.g. AT-rich, hairpin structures, T-stretch, C-rich,tandem repeats), believed to play a role in initiating and/or regulating mitochondrial transcription and replication [2], [45]–[48]. The largest non-coding region (MNR) of the six mt genomes with all the patterns mentioned above is identified as a putative control region (CR). As highly rearranged gene order in bivalves, the MNR is not conserved at the same location among bivalves [42]. In this study, four different locations (between *rrnS* and *trnM*, *trnG* and *trnI*, *cob* and *trnG*, *nad2* and *trnK*) of MNR occur.

Among the six mt genomes, the MNRs vary in size from 674 bp (*S. diphos*) to 2272 bp (*S. olivacea*) (Table 2). Moreover, the A+T content of the putative CR in each mt genome is higher or slightly lower than that of the whole mt genome. There are some sections of nucleotide sequence existed in MNR, all of which can form a typical hairpin-like secondary structures (Figure S1). The conserved flanking sequences around the hairpin structures exhibit conserved motifs: 5′-flanking sequences show a TATA element (except for *S. divaricatus*) which has also been reported in Crustacea [49], while 3′-flanking sequences possess a TTTAT element in *M. iridescens*, *S. scaba*, *S. olivacea* and *S. divaricatus*. It is assumed that these structures are of functional importance involving in the origin of the replication of mtDNA [48]. Long T-stretches of 18 bp, 15 bp, 18 bp and 13bp are observed in the MNR of *S. diphos*, *S. olivacea*, *S. divaricatus* and *S. constricta*, respectively, which may provide essential signals for the replication initiation of mtDNA [46]. In addition, the C-rich sequences, predicted to facilitate formation of the D-loop by decelerating the extension of heavy-strand synthesis at this location in some vertebrates [45], exist in the MNR of *M. iridescens*, *S. scaba* and *S. olivacea*.

Tandem repeats are also detected in MNR of five mt genomes (Figure S2; Figure S3; Figure S4; Figure S5; Figure S6; Figure S7), but that of *S. diphos*. Especially in MNR of *M. iridescens* mt genome, three distinct tandem repeat units are found, one of which comprises 14.4 nearly identical copies of a 54 bp unit. Besides, *S. olivacea* has 2 copies of 98 bp and *S. scaba* has 2.8 copies of 109 bp. Such large tandem repeat units are also reported in the bivalves *Acanthocardia tuberculata* [50] and *Placopecten magellanicus* [51]. Further study on tandem repeats in the control region is needed, as it is important to illuminate the functional implications of the repeats and the molecular mechanisms that generate the repeats [52].

### Gene order rearrangements

In contrast with other metazoans, the phylum Mollusca has long been known to exhibit an exceptionally variable arrangement of genes within mitochondrial DNA [53]–[55]. So far, due to coding on one strand probably, all bivalves whose mt genomes have been presented display enormous gene rearrangements (but see oysters [56]). It is suspected that coding on both strands may be inhibitory to mt genome rearrangement, because rearranging a genome with dual-strand coding may be more complicated and cause more harm than that codes on one strand [57].

**Figure 1:**
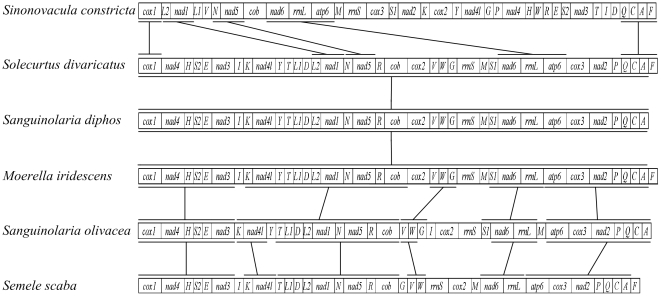
Linear representation of the mitochondrial gene arrangement in six newly sequenced bivalves. As is the standard convention for metazoan mt genomes, cox1 has been designated the start point for all genomes. All genes are transcribed from left-to-right. The bars indicate identical gene blocks. The non-coding regions are not presented and gene segments are not drawn to scale.

In this study, we compare the gene order rearrangements of six newly sequenced mt genomes (Figure 1). The six heterodont bivalves exhibit five different gene orders, among which *M. iridescens* and *S. divaricatus* have the identical gene order. Furthermore, the five patterns of gene arrangement differ from any gene order ever reported in molluscs. The gene order of *S. constricta* is remarkably unlike that of five other species, even excluding the tRNA, which might indicate the relatively distant relationship as also revealed in the phylogenetic analysis in this study (see below). And five other complete mt genomes differ primarily in the position of tRNA genes, whose secondary structures allow them to translocate more frequently than either rRNAs or protein coding genes [58], [59]. When disregarding tRNA genes, all five species belonging to superfamily Tellinoidea show the same gene arrangements except for translocations of genes *rrnS* and *cox2* in *S. scaba*. The relatively high level of conservation in the gene order may verify the close lineage relationship. In addition, there are three small blocks, *trnK*-*nad4l*, *trnV*-*trnW* and *nad6*-*rrnL*, and three large blocks, *cox1-nad4-trnH-trnS2-trnE-nad3, trnT-trnL1-trnD-trnL2-nad1-trnN-nad5-trnR-cob* and *atp6-cox3-nad2-trnP-trnQ-trnC-trnA*, shared by *S. olivacea* and *S. scaba*.

One of our noteworthy finding during this study is that *S. diphos* and *S. olivacea*, which belong to the same genus *Sanguinolaria*, have different gene arrangements with three translocations of *trnI*, *trnV*-*trnW*-*trnG* and *trnM*. The case that differences in the gene arrangement occur in the same genus is seldom reported, yet in genus *Dendropoma* [16] and genus *Crassostrea* [57]. Besides, *M. iridescens*, *S. divaricatus* and *S. diphos* have a completely identical gene order, if lacking a *trnF* in *S. diphos* is ignored. As described above, it is surprising that the gene arrangement of *S. diphos* is more similar with that of *M. iridescens* and *S. divaricatus* than that of *S. olivacea*, while *S. diphos* and *S. olivacea* should have close relatives according to traditional taxonomy. Meanwhile, this result is consistent with the conclusion from the phylogenetic analysis (see below). Early analyses of mtDNA had led to the proposition that a comparative analysis of mt gene order could proved to be a useful phylogenetic tool [11], [60], [61]. By mt gene order comparisons, Smith et al. provided evidence that two echinoderm classes, sea stars and brittle stars, form a monophyletic group to the exclusion of two others, sea cucumber and sea urchins [60]. Akasaki et al. examined the relationships of subclass Coleoidea via comparing extensive mt gene arrangements, and concluded that order Octopoda might be the most ancestral among this subclass Coleoidea in accordance with the phylogenetic tree [61]. In this study, the results obtained here support that comparisons of mt gene order rearrangments, to some extent, are a useful tool for phylogenetic studies.

### Phylogenetic analyses of Heterodonta

**Figure 2:**
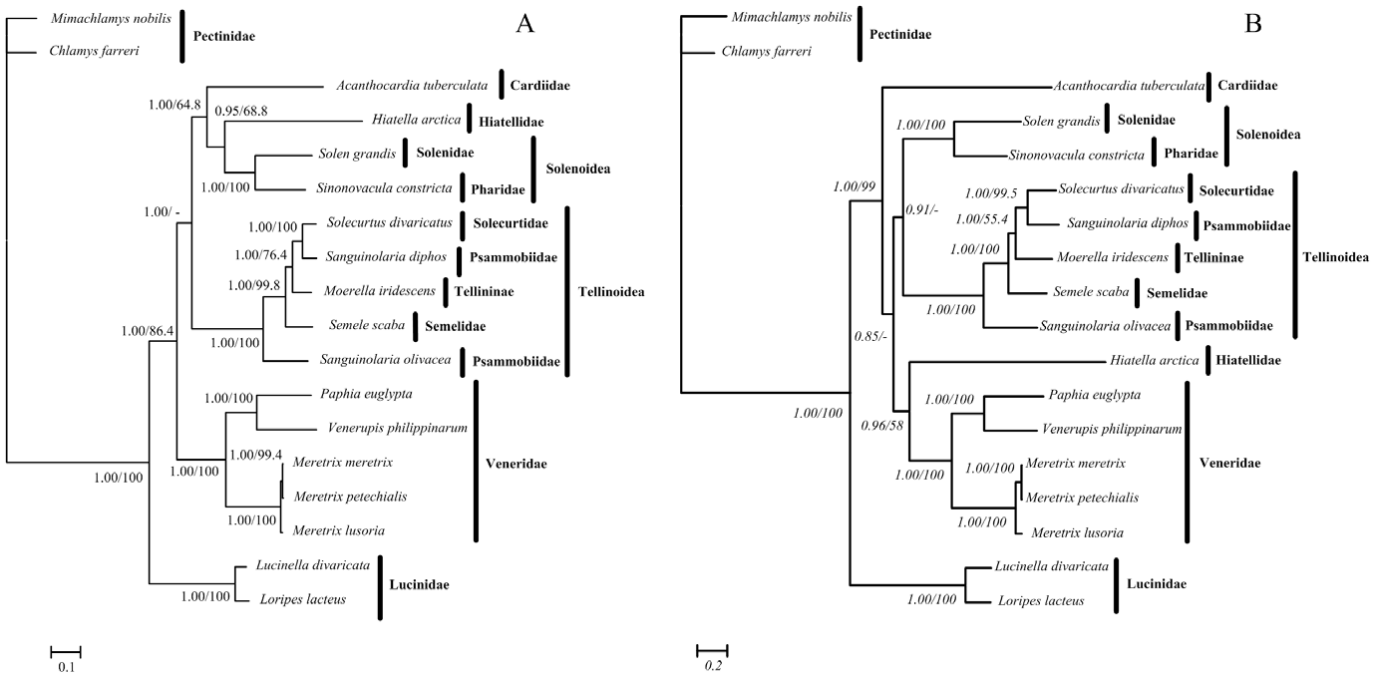
Phylogenetic trees of heterodont bivalves based on the concatenated amino acid (A) and nucleotide sequences (B) of 12 protein-coding genes (except *atp8* gene). Numbers in the nodes correspond to Bayesian posterior probabilities (left) and ML bootstrap proportions (right). Dashes indicate support values below 50%.

ML and Bayesian trees based on amino acid and nucleotide sequences of 12 concatenated protein-coding genes (except *atp8* gene) were performed to reconstruct phylogenetic relationships within heterodont bivalves (Figure 2). The tree topologies based on amino acid and nucleotide sequences were largely congruent and received high supports in most nodes.

Using mt genome data, the KH and SH tests were performed to evaluate the alternative morphology-based hypotheses and previous molecular studies. All 7 alternative topologies including 2 topologies presented in our study were summarized in Table S1. The best topology was phylogenetic analysis based on amino acid data shown in Figure 2A (KH and SH tests p = 1.000), while the second best one was phylogenetic analysis based on nucleotide sequences in Figure 2B (KH test p = 0.005 and SH test p = 0.210). All other topologies were significantly rejected (KH and SH P<0.001) which confirmed the phylogenetic analyses based on our data.

Our analysis shows the two species of family Lucinidae form a single clade as a sister group to other heterodont bivalves, indicating that Lucinidae is monophyletic, in accordance with previous viewpoint of Taylor [17]. Five species belonging to family Veneridae, including *Paphia euglypta*, *Venerupis philippinarum*, *Meritrix meretrix*, *Meritrix petechialis* and *Meretrix lusoria* cluster together, supporting the monophyly of family Veneridae [62], [63]. In the phylogenetic trees based on the amino acid data, the rock-boring and nestling *Hiatella arctica* belonging to the order Myoida is as a sister group with Solenidae+Pharidae (superfamily Solenoidea) questioning the subdivision of the subclass Heterodonta into two orders Veneroidea and Myoida, which corroborates the finding of previous molecular analysis [17], [64]. It should be noticed that *A. tuberculata* evidenced as a member of superfamily Cardioidea based on molecular analysis [17], [50], is placed in a long branch as a sister group to Hiatelloidea+Solenoidea, which is robustly supported by BI but ML based on 12-protein amino acid data (Figure 2A). While in the phylogentic analyses based on nucleotide data, the position of this species is as sister group with the other heterodont bivalves except for Lucinidae (Figure 2B). Both of results are incompatible with a sister relationship of Cardioidea to Tellinoidea, reported by Campbell [23], Steiner and Hammer [65], Dreyer et al. [66], and Taylor et al. [34] based on short fragments of nuclear gene or mt DNA. Therefore, more phylogenetic analyses based on more molecular data, in addition to morphological characters, are required in order to resolve the relationship among the subclass Heterodonta in the future.

The position of genus *Sinonovacula* has been debated over a long time. Using morphological evidences such as the formation of the siphons and so on, *Sinonovacula* had previously been suggested by Yonge [67], [68] that it should be removed from the family Solenidae where it was placed by Ghosh [69] and transferred to the Tellinoidea. This decision was accepted and followed by Keen [70], Habe [71] and Vokes [72] who transferred *Sinonovacula* to Solecurtidae within the Tellinoidea. Subsequently, using shell and anatomical characters, von Cosel [73] retransferred *Sinonovacula* to Solenoidea. And then, the result of phylogenetic analyses of heterodont bivalves based on rRNA genes by Taylor [17] was in agreement with von Cosel's decision. Our phylogenetic analyses of multiple protein-coding genes not only show that *S. constricta* is distant from the Solecurtidae belonging to Tellinoidea, but also indicate that *S. constricta* is as a sister group with *Solen grandis* of family Solenidae. In other words, we prefer classifying the genus *Sinonovacula* within the superfamily Solenoidea and not the superfamily Tellinoidea.

All five species of superfamily Tellinoidea from four different families form a clade strongly supported by three trees (except for ML tree based on amino acid sequence), which corroborates the monophyly of Tellinoidea. This result is also reported by Taylor et al. [17]. However, according to the phylogenetic trees in this study, *S. diphos* has a closer relationship with *S. divaricatus*, *M. iridescens* and *S. scaba* rather than with *S. olivacea*, highly supported by BI and ML, implying that two species presently classified into *Sanguinolaria* (Psammobiidae) do not form monophyletic groups. These unexpected findings suggesting that the current taxonomy should be brought into question and a careful review of this genus is required. Similar conclusions that Semelidae, Donacidae and Tellinidae are not monophyletic were ever made when Taylor et al. analyzed familial relationships within Tellinoidea [17]. In fact, to date, there has no special research for the taxonomy of this superfamily based on molecular data. Thereby, in the future study, more detailed analyses with a larger taxon sampling and more rapidly evolving molecular markers including mt genome are still necessary in order to test the taxonomy of superfamily Tellinoidea based on morphology and clarify familial relationships within this superfamily.

### Conclusions

In this study, we newly determined the complete mt genomes of six bivalves, increasing the number of complete mt genomes sequenced within subclass Heterodonta from 10 to 16. By comparative study of the gene order rearrangements and phylogenetic relationships of the species belonging to Tellinoidea, our results support that comparisons of mt gene order rearrangements, to some extent, are a useful tool for phylogenetic studies. Based on phylogenetic analyses of multiple protein-coding genes, we prefer classifying the genus *Sinonovacula* within the superfamily Solenoidea and not the superfamily Tellinoidea. Besides, both gene order and sequence data agree that *Sanguinolaria* (Psammobiidae) is not monophyletic. Nevertheless, more studies based on more mt genomes via combination of gene order and phylogenetic analysis are needed to further understand the phylogenetic relationships in subclass Heterodonta including superfamily Tellinoidea and Solenoidea.

## Methods

### Taxon sampling and DNA extraction

In this study, each of the six bivalve complete mt genomes sequenced was obtained from a single specimen. *M. iridescens* was collected from Leqing (Zhejiang province of China), *S. diphos* was from Beihai (Guangxi province of China), *S. scaba* was from Lingao (Hainan province of China), and meanwhile, all three were preserved in EtOH 95% in 2008. *S. olivacea* was sampled in Rizhao (Shandong province of China) and preserved frozen at −80°C in 2009. *S. constricta* and *S. divaricatus* were collected in Qingdao (Shandong province of China) and Rizhao in 2011, respectively.

The total genomic DNA was extracted from adductor muscle by a modification of standard phenol–chloroform procedure that has been described by Li et al. [74] and visualized on 1.0% agarose gel.

### PCR amplification and sequencing

In order to design long-PCR primers, we first obtained partial *cox1* and *rrnL* gene sequences, with the universal primers of LCO1490/HCO2198 [75], and 16SF/16SR [76], respectively. Polymerase chain reaction (PCR) was performed in a total volume of 50 µl including 2 U Taq DNA polymerase (Takara), about 100 ng template DNA, 1 µM forward and reverse primers, 200 µM of each dNTP, 1×PCR buffer and 2 mM MgCl_2_. The PCR reaction was carried out in TaKaRa PCR Thermal Cycler Dice Model TP600 (Takara Bio Inc.) under the following conditions: an initial denaturation for 3 min at 94°C, then 35 cycles of denaturation for 45 s at 94°C, annealing for 45 s at 52°C, extension for 1 min at 72°C, and final extension for 5 min at 72°C.

Subsequently, each mitochondrial genome was amplified by long-PCR technique [77] based on the two specific primer pairs, which were designed from the obtained partial sequences using Primer Premier 5.0 (http://www.premierbiosoft.com/). PCR reactions were carried out in 50 µl reaction mixtures containing 33.5 µl of sterile distilled H_2_O, 5 µl of 10×LA PCR buffer II (Mg^2+^ plus, Takara), 8 µl of dNTP (10 mM each), 1 µl of each primer (10 µM), 0.5 µl of LA Taq polymerase (5 U/µl, Takara), and 1 µl of DNA template (50 ng). The long PCR cycling was set up with an initial denaturation step at 94°C for 2 min, followed by 35 cycles comprising denaturatin at 94°C for 20 s, annealing at 60°C for 35 s and extension at 68°C for from 7 to 15 min depending on the expected length of the PCR products. The process was completed with a final extension at 72°C for 10 min. PCR products were purified using EZ-10 spin column DNA gel extraction kit (Sangon Biotech), and then directly sequenced with the primer walking method. The sequencing was conducted on an ABI PRISM 3730 (Applied Biosystems) automatic sequencer.

### Sequence analysis and gene annotation

All sequence data were analysed and arranged to create the full genome using the Seqman program from DNASTAR (http://www.DNASTAR.com). Protein coding genes were analysed by ORF Finder [78] using the invertebrate mitochondrial code. The rRNA genes were identified with DOGMA [79] and BLAST searches [80]. The boundaries of each gene were determined with multiple alignments of other published bivalve mitochondrial sequences. The tRNA genes were identified by DOGMA and tRNAscan-SE Search Server [81] with a COVE score cutoff of 1.0 and the invertebrate mitochondrial genetic code for secondary structure prediction. The whole mt genome sequence was tested for potentially tandem repeats by TANDEM REPEAT FINDER, Version 4.0 [82].

### Phylogenetic analyses

Eighteen molluscan mt genomes including six newly determined mt genomes, as well as those of all other heterodont bivalves, were used to illustrate the phylogenetic relationship of Heterodonta (Table 3). *Chlamys farreri* and *Mimachlamys nobilis* from the subclass Pteriomorphia served as outgroups. Owing to the fact that most bivalve species lack the atp8 gene, amino acid sequences of 12 concatenated protein-coding genes were used in phylogenetic analysis. The alignment of the amino acid sequences of each 12 mitochondrial PCGs was aligned with Clustal X [83] using default settings, followed by manual correction. After areas of dubious alignment were isolated using Gblocks [84] (default settings) and excluded from the analysis, the 12 separate amino acid sequence alignments were concatenated to a single multiple sequence alignment, which consisted of 2026 sites. The nucleotide sequence was substituted from the concatenated amino acid alignment and the final sequence consisted of 7076 sites.

**Table 3:** List of the species whose mt genome sequences were used in phylogenetic analysis in present paper.

Species	Classification	Accession Number	Reference
*Paphia euglypta*	Bivalvia; Heteroconchia; Veneroida; Veneroidea; Veneridae	GU269271	[93]
*Venerupis philippinarum*	Bivalvia; Heteroconchia; Veneroida; Veneroidea; Veneridae	AB065375	Okazaki et al., unpublished
*Meretrix meretrix*	Bivalvia; Heteroconchia; Veneroida; Veneroidea; Veneridae	GQ463598	[94]
*Meretrix lusoria*	Bivalvia; Heteroconchia; Veneroida; Veneroidea; Veneridae	GQ903339	[95]
*Meretrix petechialis*	Bivalvia; Heteroconchia; Veneroida; Veneroidea; Veneridae	EU145977	[96]
*Lucinella divaricata*	Bivalvia; Heteroconchia; Veneroida; Lucinoidea; Lucinidae	EF043342	Dreyer et al., unpublished
*Loripes lacteus*	Bivalvia; Heteroconchia; Veneroida; Lucinoidea; Lucinidae	EF043341	Dreyer et al., unpublished
*Acanthocardia tuberculata*	Bivalvia; Heteroconchia; Veneroida; Cardioidea; Cardiidae	DQ632743	[50]
*Hiatella arctica*	Bivalvia; Heteroconchia; Myoida; Hiatelloidea; Hiatellidae	DQ632742	[50]
*Solen grandis*	Bivalvia; Heteroconchia; Veneroida; Solenoidea; Solenidae	HQ703012	[97]
*Sinonovacula constricta*	Bivalvia; Heteroconchia; Veneroida; Solenoidea; Pharidae	JN398366	This study
*Moerella iridescens*	Bivalvia; Heteroconchia; Veneroida; Tellinoidea; Tellinidae	JN398362	This study
*Sanguinolaria diphos*	Bivalvia; Heteroconchia; Veneroida; Tellinoidea; Psammobiidae	JN398363	This study
*Sanguinolaria olivacea*	Bivalvia; Heteroconchia; Veneroida; Tellinoidea; Psammobiidae	JN398364	This study
*Semele scaba*	Bivalvia; Heteroconchia; Veneroida; Tellinoidea; Semelidae	JN398365	This study
*Solecurtus divaricatus*	Bivalvia; Heteroconchia; Veneroida; Tellinoidea; Solecurtidae	JN398367	This study
*Chlamys farreri*	Bivalvia; Pteriomorphia; Pectinoida; Pectinoidea; Pectinidae	EU715252	[36]
*Mimachlamys nobilis*	Bivalvia; Pteriomorphia; Pectinoida; Pectinoidea; Pectinidae	FJ415225	Xu et al., unpublished

Two methods: Maximum likelihood (ML) and Bayesian inference (BI) were used to reconstruct phylogenetic relationships of heterodont bivalves. For phylogenetic analyses based on amino acid data, MtArt+I+G evolutionary model was chosen as the best-fit model of amino acid evolution by ProtTest version 2.4 [85] at the default setting based on Akaike Information Criterion (AIC). As the MtArt evolutionary model is not available in MrBayes, the WAG model (the second best-fit model according to ProtTest) was used in Bayesian analysis. For phylogenetic analyses based on nucleotide data, the most appropriate model GTR+I+G was selected by MODELTEST [86] using the Akaike information criterion. The ML analysis was conducted with PHYML 3.0 program [87] and 1000 bootstraps were used to estimate the node reliability. BI was performed on combined database using MrBayes 3.1 [88] In the case of the Bayesian analysis, the Markov chain Monte Carlo (MCMC) were run for 5,000,000 generations (sampling every 100 generations) to allow adequate time for convergence. After omitting the first 25,000 “burnin” tree, the remaining 25,000 sampled trees were used to estimate the 50% of majority rule consensus tree and the Bayesian posterior probabilities. All phylogenetic parameters were checked with Tracer v 1.5 [89]. Alternative phylogenetic hypotheses from previous morphological and molecular studies were tested using the Kishino-Hasegawa (KH) test [90] and Shimodaira-Hasegawa (SH) test [91] implemented in CONSEL [92].

## Supporting Information

Figure S1 **Hairpin-like secondary structures in the mitochondrial putative control regions of** ***M. iridescens*** **,** ***S. scaba*** **,** ***S. divaricatus*** **,** ***S. diphos*** **,** ***S. olivacea*** **and** ***S. constricta*** **.** Conserved motifs in 5′- and 3′-flanking sequences are underlined.
(DOC)

Figure S2 **Alignment of the tandem repeats in the largest non-coding region (MNR) of mitochondrial genome of** ***Moerella iridescens*** **A.** Symbol “-” indicates an insertion or deletion.
(DOC)

Figure S3 **Alignment of the tandem repeats in the largest non-coding region (MNR) of mitochondrial genome of** ***Moerella iridescens*** **B.** Symbol “-” indicates an insertion or deletion.
(DOC)

Figure S4 **Alignment of the tandem repeats in the largest non-coding region (MNR) of mitochondrial genome of** ***Sanguinolaria olivacea*** **.** Symbol “-” indicates an insertion or deletion.
(DOC)

Figure S5 **Alignment of the tandem repeats in the largest non-coding region (MNR) of mitochondrial genome of** ***Semele scaba*** **.** Symbol “-” indicates an insertion or deletion.
(DOC)

Figure S6 **Alignment of the tandem repeats in the largest non-coding region (MNR) of mitochondrial genome of** ***Sinonovacula constricta*** **.** Symbol “-” indicates an insertion or deletion.
(DOC)

Figure S7 **Alignment of the tandem repeats in the largest non-coding region (MNR) of mitochondrial genome of** ***Solecurtus divaricatus*** **.** Symbol “-” indicates an insertion or deletion.
(DOC)

Table S1 **Tests of alternative topologies.**
(DOC)
