# Supplementary figures and images for: The Complete Mitochondrial Genomes of Six Heterodont Bivalves (Tellinoidea and Solenoidea): Variable Gene Arrangements and Phylogenetic Implications
Source: PLoS One. 2012 Feb 23;7(2):e32353. doi: 10.1371/journal.pone.0032353 (PMC3285693; doi:10.1371/journal.pone.0032353)

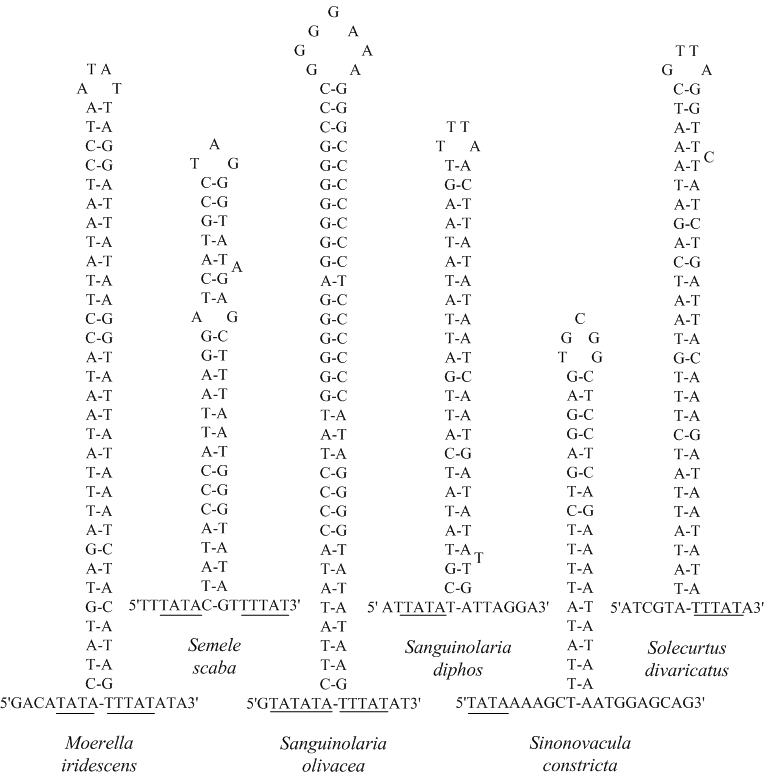

Supplement: Figure S1 — Hairpin-like secondary structures in the mitochondrial putative control regions of M. iridescens , S. scaba , S. divaricatus , S. diphos , S. olivacea and S. constricta . Conserved motifs in 5′- and 3′-flanking sequences are underlined. (DOC) [file pone.0032353.s001.doc]
