# Supplementary material for: The Complete Mitochondrial Genomes of Six Heterodont Bivalves (Tellinoidea and Solenoidea): Variable Gene Arrangements and Phylogenetic Implications
Source: PLoS One. 2012 Feb 23;7(2):e32353. doi: 10.1371/journal.pone.0032353 (PMC3285693; doi:10.1371/journal.pone.0032353)
Supplement: Table S1 — Tests of alternative topologies. (DOC) [file pone.0032353.s008.doc]

| **Alternative topologies** | **Tree topology (hypotheses)** | Rank | ln*L* | Δln*L* | KH | SH |
| --- | --- | --- | --- | --- | --- | --- |
| (1,((2,3),(((4,5),(6,(7,8))),((9,(10,(11,12))),(13,(14,(15,(16,17)))))))); | Figure 2A (Best tree) | 1 | -41761.819 | Best | 1.000 | 1.000 |
| (1,((2,3),(9,((10,((4,5),(6,(7,8)))),((11,12),(13,(14,(15,(16,17))))))))); | Figure 2B | 2 | -41856.058 | -94.239 | 0.005 | 0.210 |
| (1,(10,((2,3),((4,5),(6,7,8)),9,11,((13,16),14,15,(12,17))))); | Moore, 1969 | 6 | -43681.085 | -1919.265 | ﹤0.001 | ﹤0.001 |
| (1,(10,((2,3),((4,5),(6,7,8)),9,11,(12,(13,16),14,15,17)))); | Habe, 1977 | 7 | -43691.423 | -1929.604 | ﹤0.001 | ﹤0.001 |
| (1,((2,3),(((11,12),10),((9,((13,16),((14,15),17))),(4,5,(6,7,8)))))); | Taylor et al, 2007 | 3 | -42131.402 | -369.583 | ﹤0.001 | ﹤0.001 |
| (1,((2,3),((4,5),(6,7,8)),9,10,(11,12),((13,16),14,15,17))); | Bieler and Mikkelsen 2006 | 5 | -42445.079 | -683.26 | ﹤0.001 | ﹤0.001 |
| (1,(10,(((2,3),(9,((13,16),14,15,17))),((11,12),((4,5),(6,7,8)))))); | Giribet and Wheeler 2002 | 4 | -42146.610 | -384.791 | ﹤0.001 | ﹤0.001 |

1: *Chlamys farreri* (Outgroup) 2: *Loripes lacteus* 3: *Lucinella divaricata* 4: *Paphia euglypta* 5: *Venerupis philippinarum* 6: *Meretrix lusoria* 7: *Meretrix meretrix*

8: *Meretrix petechialis* 9: *Acanthocardia tuberculata* 10: *Hiatella arctica* 11: *Solen grandis* 12: *Sinonovacula constricta* 13: *Sanguinolaria olivacea* 14: *Semele scaba* 15: *Moerella iridescens* 16: *Sanguinolaria diphos* 17: *Solecurtus divaricatus*
