# Supplementary material for: The Complete Mitochondrial Genomes of Six Heterodont Bivalves (Tellinoidea and Solenoidea): Variable Gene Arrangements and Phylogenetic Implications
Source: PLoS One. 2012 Feb 23;7(2):e32353. doi: 10.1371/journal.pone.0032353 (PMC3285693; doi:10.1371/journal.pone.0032353)
Supplement: Supplementary file 3 [file pone.0032353.s002.doc]

Sequence positon: 10329-10371 Copynumber: 2.1
Consensus pattern (21 bp): AAGTGGTGTATATAGTATTTT

10319 TGCTACTAGA

10329 AAGTGGTGT –TAT– GTATTTT
1 AAGTGGTGTATATAGTATTTT

10348 AAGTGGTTTATATAGTATTTT
1 AAGTGGTGTATATAGTATTTT

10369 AAG
1 AAG

10372 ACAGTGGTGA

Sequence positon: 10536-10596 Copynumber: 3.0
Consensus pattern (22 bp): TATATAAATAAAGTAATAAAAG

10526 CTACATAGTT

10536 TATATAAATAAAGTAATAAAAG
1 TATATAAATAAAGTAATAAAAG

10558 TGTATAAATTAA –TAAT –AAA –
1 TATATAAATAAAGTAATAAAAG

10577 TATATATATAAA –TGATAAAA
1 TATATAAATAAAGTAATAAAA

10597 ATAGACAAAT
