# Supplementary material for: The Complete Mitochondrial Genomes of Six Heterodont Bivalves (Tellinoidea and Solenoidea): Variable Gene Arrangements and Phylogenetic Implications
Source: PLoS One. 2012 Feb 23;7(2):e32353. doi: 10.1371/journal.pone.0032353 (PMC3285693; doi:10.1371/journal.pone.0032353)
Supplement: Supplementary file 4 [file pone.0032353.s003.doc]

Sequence positon: 10745-11522 Copynumber: 14.4
Consensus pattern (54 bp):
GATGGTTGATTTAAGACGTTAATGGATTTGGAGTAGGTATATAAAGAAAGTATG

10735 ATAGTTTGTA

10745 GATGGTTGATTTGAGACGTTAATGGATTTGGAGTAGGTATATAAAGAAAGTATG
1 GATGGTTGATTTAAGACGTTAATGGATTTGGAGTAGGTATATAAAGAAAGTATG

10799 GATGGTTGATTTAAGACGTTAATGGATTTGGAGTAGGTATATAAAGAAAGTATG
1 GATGGTTGATTTAAGACGTTAATGGATTTGGAGTAGGTATATAAAGAAAGTATG

10853 GATGGTTGATTTAAGACGTTAATGGATTTGGAGTAGGTATATAAAGAAAGTATG
1 GATGGTTGATTTAAGACGTTAATGGATTTGGAGTAGGTATATAAAGAAAGTATG

10907 GATGGTTGATTTAAGACGTTAATGGATTTGGAGTAGGTATATAAAGAAAGTATG
1 GATGGTTGATTTAAGACGTTAATGGATTTGGAGTAGGTATATAAAGAAAGTATG

10961 GATGGTTGATTTAAGACGTTAATGGATTTGGAGTAGGTATATAAAGAAAGTATG
1 GATGGTTGATTTAAGACGTTAATGGATTTGGAGTAGGTATATAAAGAAAGTATG

11015 GATGGTTGATTTAAGACGTTAATGGATTTGGAGTAGGTATATAAAGAAAGTATG
1 GATGGTTGATTTAAGACGTTAATGGATTTGGAGTAGGTATATAAAGAAAGTATG

11069 GATGGTTGATTTAAGACGTTAATGGATTTGGAGTAGGTATATAAAGAAAGTATG
1 GATGGTTGATTTAAGACGTTAATGGATTTGGAGTAGGTATATAAAGAAAGTATG

11123 GATGGTTGATTTAAGACGTTAATGGATTTGGAGTAGGTATATAAAGAAAGTATG
1 GATGGTTGATTTAAGACGTTAATGGATTTGGAGTAGGTATATAAAGAAAGTATG

11177 GATGGTTGATTTGAGACGTTAATGGATTTGGAGTAGGTATATAAAGAAAGTATG
1 GATGGTTGATTTAAGACGTTAATGGATTTGGAGTAGGTATATAAAGAAAGTATG

11231 GATGGTTGATTTAAGACGTTAATGGATTTGGAGTAGGTATATAAAGAAAGTATG
1 GATGGTTGATTTAAGACGTTAATGGATTTGGAGTAGGTATATAAAGAAAGTATG

11285 GATGGTTGATTTAAGACGTTAATGGATTTGGAGTAGGTATATAAAGAAAGTATG
1 GATGGTTGATTTAAGACGTTAATGGATTTGGAGTAGGTATATAAAGAAAGTATG

11339 GATGGTTGATTTAAGACGTTAATGGATTTGGAGTAGGTATATAAAGAAAGTATG
1 GATGGTTGATTTAAGACGTTAATGGATTTGGAGTAGGTATATAAAGAAAGTATG

11393 GATGGTTGATTTAAGACGTTAATGGATTTGGAGTAGGTATATAAAGAAAGTATG
1 GATGGTTGATTTAAGACGTTAATGGATTTGGAGTAGGTATATAAAGAAAGTATG

11447 GATGGTTGATTTGAGACGTTAATGGATTTGGAGTAGGTATATAAAGAAAGTATG
1 GATGGTTGATTTAAGACGTTAATGGATTTGGAGTAGGTATATAAAGAAAGTATG

11501 GATGGTTGATTTGAGACGTTAA
1 GATGGTTGATTTAAGACGTTAA

11523 CGATTAGCCA
