# Supplementary material for: The Complete Mitochondrial Genomes of Six Heterodont Bivalves (Tellinoidea and Solenoidea): Variable Gene Arrangements and Phylogenetic Implications
Source: PLoS One. 2012 Feb 23;7(2):e32353. doi: 10.1371/journal.pone.0032353 (PMC3285693; doi:10.1371/journal.pone.0032353)
Supplement: Supplementary file 5 [file pone.0032353.s004.doc]

Sequence position: 10594-10794 Copynumber: 2.0
Consensus pattern (98 bp):
GGGGTATTTTTATTTTATAGTTAGAGATATTAAGCTTATACCTTACTTTATTTGTAAAAATTGTA
AAAAATTACGAAAAAAGAAATTATTTATAATTA

10584 TATCGATTTT

10594 GGGGTATTTTTATTTTATAGTTAGAGATATTAAGCTTATGCCTTACTTTATTTGTAAAAATTGTA
1 GGGGTATTTTTATTTTATAGTTAGAGATATTAAGCTTATACCTTACTTTATTTGTAAAAATTGTA

10659 AAAAATTATGAAAAAAGGCC–TTTTTTATCAATTC
66 AAAAATTACGAAAAAAGAAATTATTTAT – AATTA

10693 GGGGTATTTTTATTTTATAGTTAGAGATATTAAGCTTATACCTTATTTTATTTGTAAAAATTGTA
1 GGGGTATTTTTATTTTATAGTTAGAGATATTAAGCTTATACCTTACTTTATTTGTAAAAATTGT–

10758 AAAAAATTACG– GAAATGAAATTATTTATAATTA
65 AAAAAATTACGAAAAAAGAAATTATTTATAATTA

10791 GGGG
1 GGGG

10795 AAGTTTGTAT
