# Supplementary material for: The Complete Mitochondrial Genomes of Six Heterodont Bivalves (Tellinoidea and Solenoidea): Variable Gene Arrangements and Phylogenetic Implications
Source: PLoS One. 2012 Feb 23;7(2):e32353. doi: 10.1371/journal.pone.0032353 (PMC3285693; doi:10.1371/journal.pone.0032353)
Supplement: Supplementary file 6 [file pone.0032353.s005.doc]

Sequence position: 8871-8940 Copynumber: 6.0
Consensus pattern (11 bp): AAATAAAAAAT

8861 CGTAGTAATA

8871 AAATAAAAAAT
1 AAATAAAAAAT

8882 AAATAAAAAAT
1 AAATAAAAAAT

8893 AAATAAAAAAAAT
1 AAAT – –AAAAAAT

8906 AAATAAAAAAAAT
1 AAAT – –AAAAAAT

8919 AAATAAAAAAT
1 AAATAAAAAAT

8930 AAATAAAAAAT
1 AAATAAAAAAT

8941 GAAGAACTGA

Sequence position: 9241-9550 Copynumber: 2.8
Consensus pattern (109 bp):
GATTTCTGTGGGGGGGGGGGTAAACGATAAGGGCAAAATTCGGTAGGGAGATAGGGGCACTTTAG
TGGAAATCGGATAGTAGTAAAAAGAAAATGGGGGCGTCGGCCAG

9231 TGGGGGGAGA

9241 GATTTCTGTGGGGGGGGGGGTAAACGACAAGGGCAAAATTCGGTAGGGAGATAGGGGCACTTTAG
1 GATTTCTGTGGGGGGGGGGGTAAACGATAAGGGCAAAATTCGGTAGGGAGATAGGGGCACTTTAG

9306 TGGGAATCGGATAGTAGTAAAAAGAAAATGGGGGCGTCGGCCAG
66 TGGAAATCGGATAGTAGTAAAAAGAAAATGGGGGCGTCGGCCAG

9350 GATTTCTGCAGGGGGGGGGGGTAAACGATAAGGGCAAAATTCGGCAGGGAGATAGGGGCACTTTAG
1 GATTTCTGTGGGGGGGGGGGTAAACGATAAGGGCAAAATTCGGTAGGGAGATAGGGGCACTTTAG

9415 TGGAAATCGGATAGTAGTAAAAAGAAAATGGGGGCGTCGGCCAG
66 TGGAAATCGGATAGTAGTAAAAAGAAAATGGGGGCGTCGGCCAG

9459 GATTTCTGTGGGGGGGGGGGTAAACGATAAGGGCAAAATTCGGTAGGGAGATAGGGGCACTTTAG
1 GATTTCTGTGGGGGGGGGGGTAAACGATAAGGGCAAAATTCGGTAGGGAGATAGGGGCACTTTAG

9524 TGGAAATCGGATAGTAGTAAAAAGAAA
66 TGGAAATCGGATAGTAGTAAAAAGAAA

9551 CCGAGGGGGC
