# Supplementary material for: The Complete Mitochondrial Genomes of Six Heterodont Bivalves (Tellinoidea and Solenoidea): Variable Gene Arrangements and Phylogenetic Implications
Source: PLoS One. 2012 Feb 23;7(2):e32353. doi: 10.1371/journal.pone.0032353 (PMC3285693; doi:10.1371/journal.pone.0032353)
Supplement: Supplementary file 7 [file pone.0032353.s006.doc]

Sequence position: 12753-12782 Copynumber: 1.9
Consensus pattern (16 bp): GAAGAAAAAATCCTGG

12743 CATAATATAA

12753 GAAGAAAAAATCCTGG
1 GAAGAAAAAATCCTGG

12769 GAAG– AAAAATCCTG
1 GAAGAAAAAATCCTG

12783 AAAGCTAATA
