# Supplementary material for: The Complete Mitochondrial Genomes of Six Heterodont Bivalves (Tellinoidea and Solenoidea): Variable Gene Arrangements and Phylogenetic Implications
Source: PLoS One. 2012 Feb 23;7(2):e32353. doi: 10.1371/journal.pone.0032353 (PMC3285693; doi:10.1371/journal.pone.0032353)
Supplement: Supplementary file 8 [file pone.0032353.s007.doc]

Sequence position: 10563-10593 Copynumber: 2.5
Consensus pattern (13 bp): ATATATGTGTATA

10553 TCTATATATC

10563 ATATATGTGTATA
1 ATATATGTGTATA

10576 ATATATGTGTA–A
1 ATATATGTGTATA

10588 ATATAT
1 ATATAT

10594 AATACACATA
